# An Integrated LC-MS-Based Strategy for the Quality Assessment and Discrimination of Three *Panax* Species

**DOI:** 10.3390/molecules23112988

**Published:** 2018-11-15

**Authors:** Zhixia Du, Jinhua Li, Xiang Zhang, Jin Pei, Linfang Huang

**Affiliations:** 1 Chengdu University of Traditional Chinese Medicine, Chengdu 611137, China; zxdu712@126.com; 2 Key Research Laboratory of Traditional Chinese Medicine Resources Protection, Administration of Traditional Chinese Medicine, National Administration of Traditional Chinese Medicine, Institute of Medicinal Plants, Chinese Academy of Medical Sciences, Beijing 100193, China; pjcdzy03@163.com (J.L.); 18364166427@163.com (X.Z.)

**Keywords:** qualitative analysis, quantitative analysis, ginsenosides, panax species, UHPLC-Q-Exactive/HRMS

## Abstract

The quality assessment and discrimination of Panax herbs are very challenging to perform due to the complexity and variability of their chemical compositions. An integrated strategy was established using UHPLC-Q-Exactive/HRMS and HPLC-ESI-MS/MS to achieve an accurate, rapid, and comprehensive qualitative and quantitative analysis of *Panax japonicas* (PJ), *Panax japonicus* var. major (PM), and *Panax zingiberensis* (PZ). Additionally, discrimination among the three species was explored with partial least squares–discriminant analysis (PLS-DA) and orthogonal partial least squares–discriminant analysis (OPLS-DA) score plots. A total of 101 compounds were plausibly or unambiguously identified, including 82 from PJ, 78 from PM, and 67 from PZ. Among them, 16 representative ginsenosides were further quantified in three herbs. A clear discrimination between the three species was observed through a multivariate statistical analysis on the quantitative data. Nine compounds that allowed for discrimination between PJ, PM, and PZ were discovered. Notably, ginsenoside Rf (G-Rf), ginsenoside F3 (G-F3), and chikusetsu saponin IV (CS-IV) were the three most important differential compounds. The research indicated that the integrated LC-MS-based strategy can be applied for the quality assessment and discrimination of the three Panax herbs.

## 1. Introduction

Ginseng herbs, the roots and rhizomes of the *Panax* species (*Araliaceae*), are valuable traditional herbs that have a thousand years of medical history and are well-known worldwide as herbal medicines and food to enhance body strength, prevent exhaustion, and improve immunity [1]. *Panax* species are the important medicinal resources in the world because of their significant medicinal value, and are distributed in more than 35 countries, particularly America, South Korea, Japan, and China. With their popularity all over the world, the attention that *Panax* has received has increased significantly [2,3,4,5]. These herbs contain complex chemical constituents. Ginsenosides are the characteristic and principal components and have been used as an important index in quality assessment and control. The skeleton of aglycones reveals dammarane tetracyclic triterpenoidal saponins and oleanane pentacyclic triterpenoidal saponins (oleanolic acid-type ginsenosides, OAs) in natural ginsensides. Furthermore, dammarane tetracyclic triterpenoidal saponins can be divided into protopanaxatiol-type ginsenosides (PPTs) and protopanaxadiol-type ginsenosides (PPDs) according to the study of dammarane structures [6]. Although numerous analytical approaches have been applied for the qualitative and quantitative analysis of ginsenosides, including UV, IR, TLC, HPLC, and UPLC, the analysis of ginsenosides is still a great challenge because of the diversity, similarity, and complexity of their chemical structures [1].

In recent years, some advanced analytical techniques have been rapidly developed for the quality assessment and control of traditional herbs. Q-Exactive Orbitrap High-Resolution Mass Spectrometry (Q-Exactive/HRMS) is a recently developed technique with extremely high resolution, sensitivity, and mass accuracy. It exhibits a stronger power for the scanning and identification of complex compounds than normal mass spectrometry [7]. LC-HRMS is now a well-established technique for the qualitative analysis of chemical compounds in metabolomic studies, and has seen significant progress in recent years [1]. Combining UHPLC with Q-Exactive/HRMS has been increasingly used to screen and identify complex compounds in herbs and food products [8,9]. Furthermore, high-performance liquid chromatography coupled with electrospray ionization tandem mass spectrometry (HPLC-ESI-MS/MS) is also a powerful tool for complex composition analysis for the quantitation and screening of specific chemicals in foods and plants [10,11,12,13]. The MS detector can provide information on molecular formula and fragmentation ions. Moreover, the multiple reaction monitoring (MRM) mode is highly specific and sensitive; it is useful for quantifying complex compounds in natural products [14].

Previous studies show that more than 10 species of the genus *Panax* are available around the world. Among them, *Panax ginseng*, *Panax quinquefolius*, and *Panax notoginseng* are the most recognized species and have been subjected to extensive study [15,16]. The other Panax species are less known. *Panax japonicas* (PJ), *Panax japonicus* var. major (PM), and *Panax zingiberensis* (PZ) are three different ginseng herbs with similar bioactivities; they grow in the mid- and low-latitude areas in the Northern hemisphere [17,18]. Similar to other Panax species, they contain a high amount of various chemical constituents, such as saponins, polysaccharides, amino acids and microelements, which are believed to contribute to their multiple bioactivities and restorative functions. PJ is widely used as a traditional medicine in China and Japan and has been recorded in the Chinese Pharmacopoeia and the Japanese Pharmacopoeia [17,19,20]. PM, which is thought to be a variety of PJ, has also been recorded in the Chinese Pharmacopoeia [18]. PZ is widely used for strengthening the immune response and providing cardiovascular protection in folk medicines of China and Myanmar [21]. At present, these three ginseng herbs are widely incorporated into health products and dietary supplements for their related and similar pharmacological functions. However, they display certain differences in their activities and effects. Currently, there are few studies on the quantitative and qualitative analysis of the chemical compositions and investigations on the differences between these three Panax herbs. Therefore, it is crucial to study their chemical compositions, perform a quality assessment, and investigate their differences.

In the present study, a new, rapid, and sensitive UHPLC-Q-Exactive/HRMS analysis method has been developed for the first time to comprehensively screen and identify the chemical compositions of the three ginseng herbs PJ, PM, and PZ. A sensitive and practical HPLC-ESI-MS/MS method was established to simultaneously separate and accurately determine the 16 major ginsenosides in 50 batches of the three ginseng samples. Finally, discrimination of the three Panax herbs and the important compounds were investigated using the partial least squares–discriminate analysis (PLS-DA) and orthogonal partial least squares–discriminant analysis (OPLS-DA) methods based on quantitative data.

## 2. Materials and Methods

### 2.1. Materials and Reagents

All ginseng samples are listed in Table 1. Sample materials were collected from China and Myanmar from 2015 to 2016. All samples were authenticated by one of the authors, Professor Linfang Huang, and were identified as the radices of *P. japonicus* C. A. Mey (PJ), *P. japonicus* C. A. Mey. var. major (Burk) C. Y. Wu et K. M. Feng (PM), and *P. zingiberensis* C. Y. Wu et K. M. Feng (PZ). The voucher specimens have been deposited at the Herbarium of the Chinese Academy of Medical Science and the Peaking Union Medicinal College.

**Figure 1:**
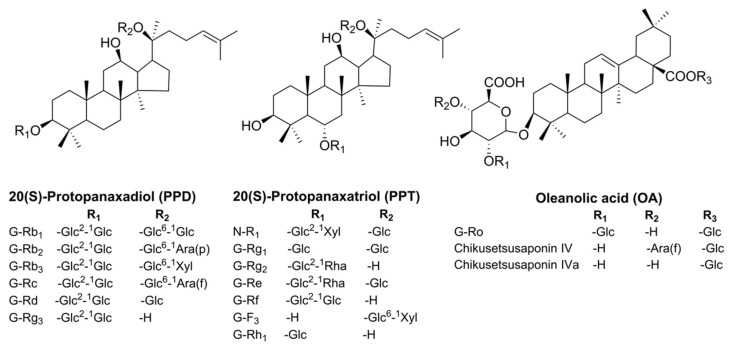
The chemical structures of the 16 reference standards.

The 16 reference standards of the ginsenosides, that is, ginsenoside (G)-Rg1, G-Re, Rb1, G-Rc, G-Rb2, G-Rb3, G-Rf, G-Rd, G-Rg2, G-Rg3, G-F3, G-Rh1, G-Rh2, G-Ro, notoginsenoside (NG)-R1, and pseudoginsenoside (pseudo G)-F11, were purchased from Chengdu Must Biotechnology Co., Ltd. (Chengdu, Sichuan, China). The four reference standards of the ginsenosides, that is, G-F1, G-F2, protopanoxadiol (PPD), and protopanaxatriol (PPT), were supplied by the National Institute of Control of Pharmaceutical and Biological Products (Beijing, China). Lastly, two reference standards of chikusetsu saponin (CS), that is, CS-IV and CS-IVa, were obtained from Chengdu Chroma-Biotechnology Co., Ltd. (Sichuan, China). The chemical structures of the 16 quantitative ginsenosides are shown in Figure 1. The purities of all reference standards were higher than 98%, as confirmed by HPLC.

LC-MS grade acetonitrile and methanol were obtained from Fisher Scientific (Beijing, China). De-ionized water was purified using a Milli-Q Ultra-pure water system (Millipore, Bedford, MA, USA). All other reagents and chemicals were of analytical grade and obtained from Beijing Chemical Plant Co. Ltd. (Beijing, China).

### 2.2. Sample Solutions Preparation

The samples of PJ, PM, and PZ were pulverized into fine powder in a grinder; 1.0 g of the powder was suspended in 25 mL of methanol and ultrasonically extracted (40 kHz, 200 W) for 30 min at 40 °C. The extracted solutions were then filtered through 0.22 μm Nylon micropore membranes and used for the UHPLC-Q-Exactive/HRMS analysis.

Furthermore, 0.1 g of the powder was suspended in 20 mL of 60% methanol and ultrasonically extracted (40 kHz, 200 W) for 45 min at 40 °C. The extracted solutions were filtered and diluted 80 times using 60% methanol. The diluted solutions were filtered through 0.22 μm Nylon micropore membranes and used for the HPLC-ESI-MS/MS analysis.

### 2.3. Standard Ginsenosides Solutions

Certain amounts of G-Rg1, G-Re, G-Rb1, G-Rc, G-Rb2, G-Rb3, G-Rf, G-Rd, G-Rg2, G-Rg3, G-F3, G-Rh1, G-Rh2, G-Ro, G-F1, G-F2, PPD, PPT, NG-R1, Pseudo G-F11 CS-IV, and CS-IVa were dissolved in methanol to obtain 22 reference compound stock solutions (at 1.0–5.0 mg/mL). The stock solutions were diluted with methanol, and each solution was used for studying fragmentation pathways by a UHPLC-Q-Exactive/HRMS analysis.

Furthermore, a mixed solution containing the references of G-Rg1, G-Re, G-Rb1, G-Rc, G-Rb2, G-Rb3, G-Rf, G-Rd, G-Rg2, G-Rg3, G-F3, G-Rh1, G-Ro, NG-R1, CS-IV, and CS-IVa was also prepared and serially diluted with 60% methanol–water (*v*/*v*) to obtain 16 reference solutions with different concentrations, which were used for plotting standard curves in the HPLC-ESI-MS/MS analysis.

### 2.4. UHPLC-Q-Exactive Orbitrap HRMS Conditions for Qualitative Analysis

UHPLC analysis was performed using an Ultimate 3000 system (Dionex, Sunnyvale, CA, USA), which is equipped with an online vacuum degasser, a quaternary pump, an autosampler, and a thermostated column compartment. An ACQUITY UPLC HSS T3, 2.1 mm × 100 mm, 1.7 μm (Waters, Milford, MA, USA) was used for chromatographic separation at 40 °C. For separation, gradient elution using aqueous formic acid 0.1% (*v*/*v*) was done for mobile phase A and acetonitrile for phase B at a flow rate of 0.3 mL/min. The following gradient was applied: 0–1 min, 0% B; 1–10 min, 0%→100% B and 10–10.1 min, 0% B. The injection volume was 2 μL, and the injection temperature was set at 15 °C.

High-Resolution Mass spectrometry was performed with a Q-Exactive Orbitrap HRMS (Thermo Fisher, Waltham, MA, USA) using a heated electrospray ionization source (HESI) for the ionization of the target compounds in the negative mode. The operating parameters were as follows: spray voltage, 3.70 KV; capillary temperature, 320 °C; sheath gas pressure, 30 psi; auxiliary gas pressure, 10 arb; auxiliary gas heater temp, 300 °C; scan modes, full MS scan (resolution 70,000); and scan range, *m*/*z* 100–1500. The data were processed using the Thermo Xcalibur 3.0 software (Thermo Finnigan, San Jose, CA, USA).

### 2.5. HPLC-ESI-MS/MS Conditions for Quantitative Analysis

For quantitative analysis, the separation of the multicomponents was carried out using an Agilent 1260 Infinity liquid chromatography (Agilent, Lexington, MA, USA) equipped with a quaternary pump, an online vacuum degasser, an autosampler, and a thermostatic column compartment. Chromatographic separation was performed on a Waters C18 column (3.9 mm × 150 mm, 4.6 μm). The mobile phase consisted of (A) acetonitrile and (B) a 0.05% formic acid aqueous solution by gradient elution (0–3 min, 20%→23% A; 3–8 min, 30%→35% A; 8–15 min, 35% A; 15–20 min, 35%→60% A; 20–22 min, 60%→80% A; 22–24 min, 80%→95% A; 24–25 min, 95%→20% A). The flow rate was 1 mL/min, and the split ratio was set at 3:2. The temperature was set at room temperature. The injection volume was 10 μL.

The Applied Biosystems 3200QTRAP triple quadrupole tandem mass spectrometer (Applied Biosystems/MDS Sciex, Concord, Ontario, Canada) used was equipped with an electrospray ionization (ESI) source for the mass analysis and detection. All data collected were analyzed and processed using the Analyst 1.6 software (Applied Biosystems/MDS Sciex. Foster City, CA, USA). The turbo ion spray source was set in the negative ionization mode. Multiple reaction monitoring (MRM) was used for detection transitions. The selective ion-pair, DP, and eV of the 16 saponins are shown in Table 2. The ion spray voltage was set at −4500 V, the source temperature was set at 450 °C, and gas 1 and gas 2 were set at 50 psi and 45 psi, respectively.

### 2.6. Quantitative Method Validation

To verify linearity, the standard solutions containing 16 reference substances at seven different concentrations were injected into the triple quadrupole tandem mass spectrometer and analyzed. Calibration curves of the reference standards were constructed by plotting the integrated peak area versus the corresponding concentrations. The limit of detection (LOD) and the limit of quantification (LOQ) of the 16 ginsenosides were determined by injecting a series of standard solutions until the basis of response at the signal-to-noise ratio (S/N) was about 3 times for LOD and 10 times for LOQ. To determine precision, the samples were analyzed six times within the same day. Reproducibility was evaluated by extracting and analyzing six replicates of the same batch of sample with the established method. To determine stability, the same sample solution was analyzed at 0, 2, 4, 8, 12, and 24 h. Furthermore, a recovery test was used to evaluate the accuracy of this method. Known amounts of ginsenoside standards were added into a 0.1 g of sample six times; the six mixtures were extracted and analyzed.

### 2.7. Multivariate Statistical Analysis

A multivariate statistical analysis was performed using the SIMCA-P 13.0 software (Umetrics AB, Umea, Sweden) using a partial least squares–discriminant analysis (PLS-DA) and an orthogonal partial least squares–discriminant analysis (OPLS-DA). The score plots of all samples employed UV scaling. The heat map was drawn using the Heatmap illustrator 1.0 (Wuhan, Hubei, China).

## 3. Results and Discussion

### 3.1. Identity Assignment and Confirmation of the Components in PJ, PM, and PZ

**Figure 2:**
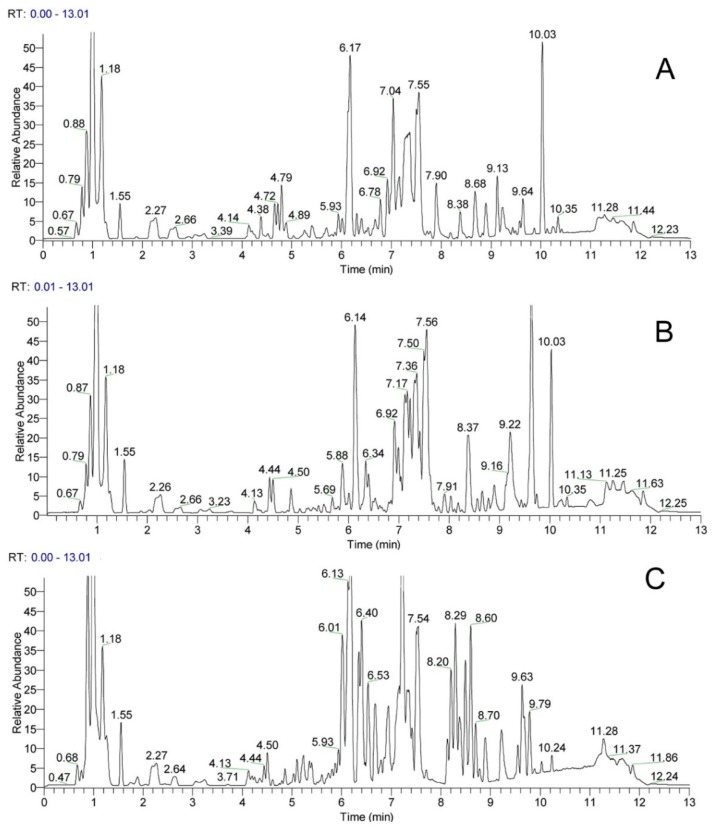
The base peak chromatogram of the roots and rhizomes of PJ (**A**), PM (**B**), and PZ (**C**) in negative mode. *Panax japonicas* (**PJ**), *Panax japonicus* var. major (**PM**), *Panax zingiberensis* (**PZ**).

In this study, the UHPLC-Q-Exactive/HRMS technique was utilized to rapidly separate and comprehensively identify the major compounds in PJ, PM, and PZ. To separate most of the compounds and achieve the most sensitive detection in a short time, the chromatographic and spectral conditions were optimized. Because the negative mode was more sensitive than the positive mode for detecting ginsenosides in the pre-experiment, heated electrospray ionization of the chemical compounds was performed in the negative ion mode. In addition, formic acid, when added to the mobile phase, not only improved the chromatographic peaks, but also easily generated formic acid adductions [M + HCOO]^−^, which made it easier to detect and confirm the molecular ion. After optimizing the experimental conditions, the base peak chromatograms were obtained as shown in Figure 2. The details for the identified ginsenosides, such as the retention time (tR), the molecular formula, the theoretical molecular mass, the experimental molecular mass, and MS/MS (fragment ion) information, are summarized in Table 3. These provide abundant information that can be used as a basis for identifying the constituents in the three Panax herbs. The mass error for molecular ions in all identified ginsenosides was within 10 ppm, indicating that the experimental molecular formula well-matched with the quasimolecular ions, theoretical molecular ions, and fragment ions.

Phytochemistry research studies have demonstrated that the chemical constituents in the *Panax* genus are very complex [1,22,23]. Ginsenosides are the major effective components. Currently, hundreds of ginsenosides have been isolated and unambiguously characterized from these *Panax* species, especially from *P. ginseng*, *P. quinquefolium*, and *P. notoginseng*. Furthermore, the fragmentation pathways of numerous ginsenosides have been reported by numerous research studies [24,25], which makes it easier to detect and identify secondary metabolites from *Panax* species using the UHPLC-Q-Exactive/HRMS technique. In the present study, ESI-MS on negative ion mode was used for compound detection and characterization due to its high sensitivity and sensitivity, as well as clear mass spectra in the negative ion mode. A total of 101 compounds were detected and tentatively identified, including 82 from PJ, 78 from PM, and 67 from PZ. Among them, 22 ginsenosides, including ginsenosides G-Rg1, G-Re, G-Rb1, G-Rc, G-Rb2, G-Rb3, G-Rf, G-Rd, G-Rg2, G-Rg3, G-F3, G-Rh1, G-Rh2, G-Ro, G-F1, G-F2, NG-R1, pseudo G-F11, PPD, PPT, CS-IV, and CS-IVa, were unambiguously identified by comparing their retention times and fragment ions with the reference standards. The others were tentatively assigned by the empirical molecular formula, theoretical molecular mass, and MS/MS fragment ions as well as the retention sequence of isomeric ginsenosides.

**Figure 3:**
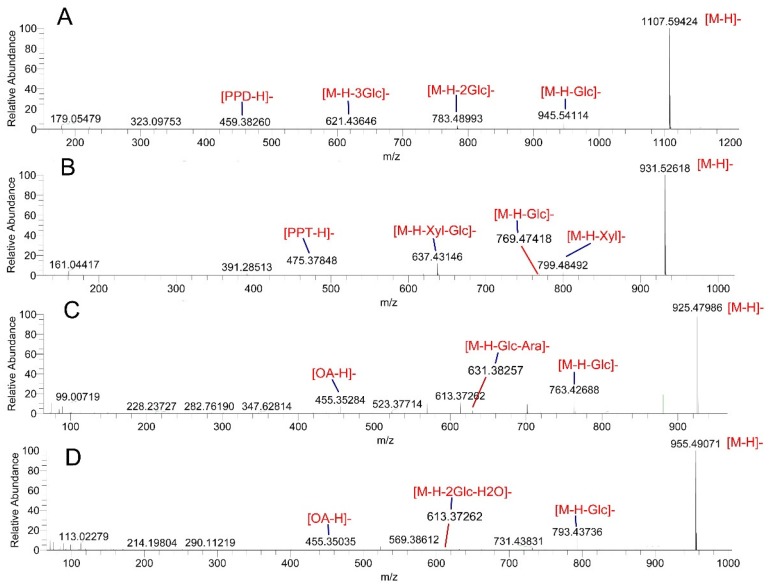
The MS/MS spectrum of ginsenoside -Rb1 (**A**), ginsenoside -R1 (**B**), ginsenoside -Ro (**C**), and chikusetsu saponin -IV (**D**).

In the MS spectra, most of the ginsenosides showed deprotonated ions [M − H]^−^ and/or formic acid adduct ions [M + HCOO]^−^ in the negative ion mode. However, it is worth noting that the malonyl-ginsenosides could not produce adduct ions [M + HCOO]^−^ because malonyl-ginsenosides are unstable. It is easy to lose CO_2_ from the deprotonated molecules of these compounds under demalonylation, which results in the detection of the peaks in the quasi-molecular ions of [M − H − CO2]^−^ and [M − H − Malonyl]^−^, which were found in compounds **19**, **22**, **31**, **40**, **45**, **46**, **50**, **51**, **56**, **57**, **59**, **65**, and **68**. Additionally, according to the negative MS, MS/MS spectra of the ginsenosides, the deprotonated ions and their product ions exhibited the common fragmentation pattern that corresponds to the successive or simultaneous loss of glycosidic units, such as the glucosyl (Glc) group (162 Da), the rhamnosyl (Rha) group (146 Da), the xylose(Xyl)/arabinose(Ara) group (132 Da), and the glucuronyl (Glu A) group (176 Da), at the C-20, C-3, or C-6 sites until the formation of an aglycone ion. The characteristic ions at *m*/*z* 475 (C_30_H_51_O_4_) and *m*/*z* 459 (C_30_H_51_O_3_) were observed in the PPTs (such as compounds **3**, **6**, **8**, **10**, **11**, **13**, **17**, **18**, **41**, **53**, **62**, **69**, and **78** ) and PPDs (such as compounds **20**, **30**, **31**, **35**, **36**, **39**, **42**, **48**, **54**, **60**, **64**, **70**, **71**, **75**, **85**, **88**, **89**, **91**, **93**, and **98** ), respectively. As shown in Figure 3A,B, G-Rb1 produced [(20*S*)-protopanaxadiol − H]^−^ at *m*/*z* 459.38260 (C_30_H_51_O_3_) in the MS/MS spectrum by the successive loss of four Glc (162 Da), and NG-R1 gave the [(20*S*)-protopanaxatriol − H]^−^ at *m*/*z* 475.37848 (C_30_H_51_O_4_) via the successive elimination of one Xyl and two Glc. Moreover, the OAs displayed an aglycone ion at *m*/*z* 455 (C_30_H_47_O_3_) corresponding to [oleanolic acid − H]^−^, which was visible for compounds **47**, **55**, **61**, **67**, **80**, **81**, **82**, **84**, and **94**. Figure 3C,D show the MS/MS spectra of G-Ro (55) and CS-IV (61). These two saponins produced [oleanolic acid − H]^−^ at *m*/*z* 455 (C_30_H_47_O_3_) after the successive loss of Glc, and/or Ara, and Glu A.

Notably, some Octillol-type triterpenoid saponins were also detected and identified in the three ginseng herbs. For example, compounds **24** (Pseudo G-F11), **34** (Vina G-R1), **95** (Vina G-R2), **100** (Pseudo G-RT4), **5**, and **21** (Vina G-R6/Yesanchinoside C) were observed at *m*/*z* 491 (C_30_H_51_O_5_) via the loss of a different sugar moiety, corresponding to the deprotonated ions of Octillol-type aglycone.

### 3.2. Validation of the Quantitative Analytical Method

The HPLC-ESI-MS/MS quantitative analysis method was validated. The regression equations, coefficient of determination, linear ranges, LODs, and LOQs for the quantitative analysis of the 16 reference ginsenosides are shown in Table 4. The calibration curves for all 16 reference substances showed good linear regression (r > 0.999) within the test ranges. The LODs of the 16 reference compounds were estimated to be 0.13–2.22 ng/mL, whereas the LOQs were 0.31–5.90 ng/mL. The precision, repeatability, stability, and recovery are listed in Table 5. The precision of the quantitative method was determined; the validation studies showed that the relative standard deviation (RSD) was less than 4.87%, and the repeatability of the method was very good (RSD < 4.93%). The RSD of the storage stability was less than 4.60% in 24 h. The recovery was in the range of 99.25–104.10% with an RSD of less than 3.45%. The established HPLC-ESI-MS/MS method was accurate and reliable and is therefore appropriate for quantitative analysis.

### 3.3. Determination of the 16 Ginsenosides Using HPLC-ESI-MS/MS

**Figure 4:**
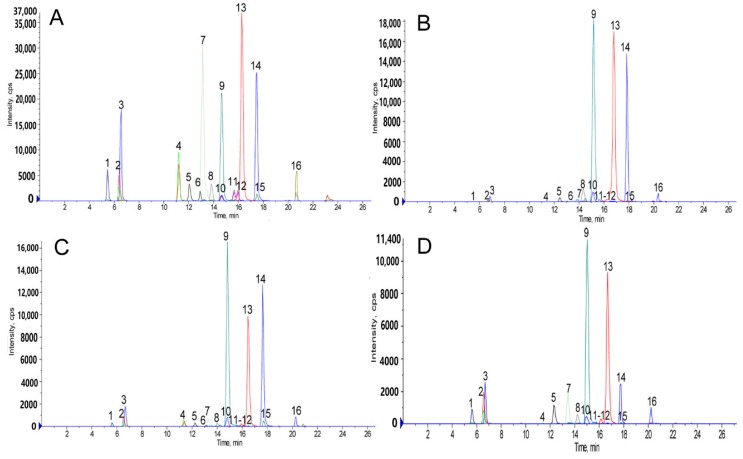
The total ion MRM chromatograms of the reference standards (**A**) and the roots and rhizomes of *Panax japonicas* (**B**), *Panax japonicus* var. major (**C**), and *Panax zingiberensis* (**D**) obtained in negative mode by HPLC-ESI-MS/MS.

**Figure 5:**
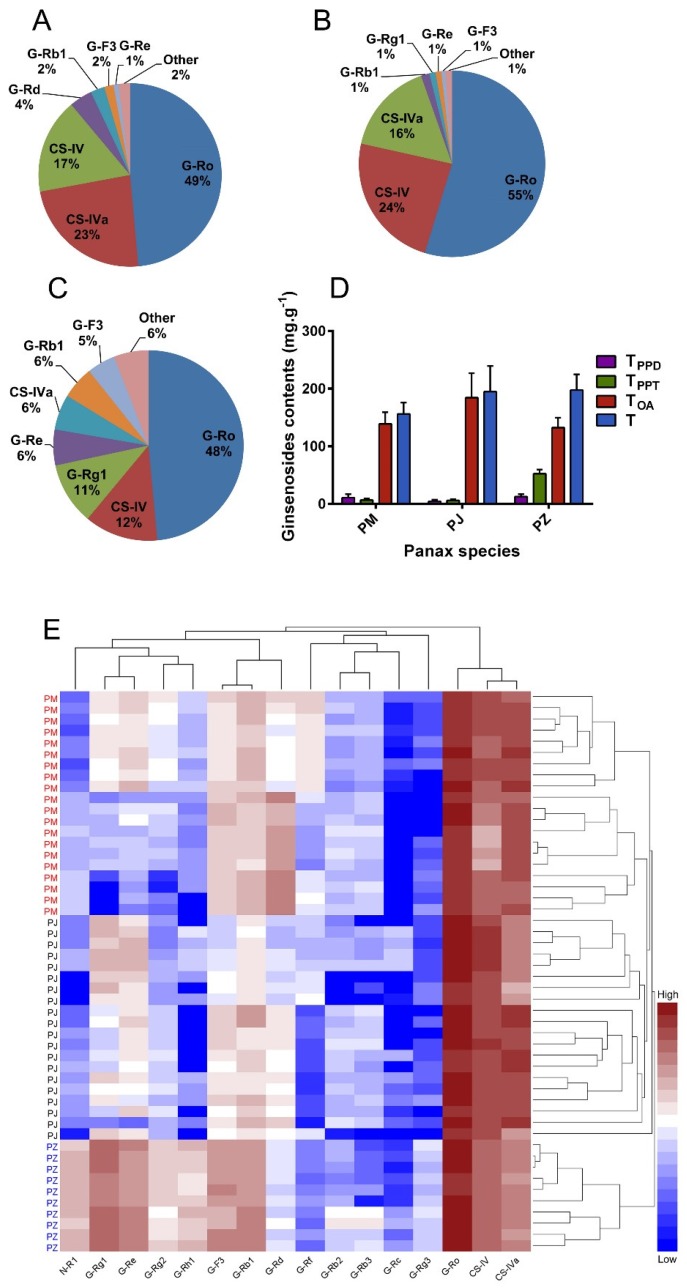
The percentage contents of the 16 ginsenosides (**A**, PJ; **B**, PM; **C**, PZ) and total ginsenosides (**D**) in the three ginseng herbs; The content difference of 16 saponins by a two-way hierarchical clustering analysis heat map (**E**). Ginsenoside (G)-Rg1, G-Re, G-Rb1, G-Rc, G-Rb2, G-Rb3, G-Rf, G-Rd, G-Rg2, G-Rg3, G-F3, G-Rh1, G-Ro. Notoginsenoside (N)-R1 and chikusetsu saponin (CS) -IV and CS-Iva.

The contents of the major 16 ginsenosides from 50 batch samples were determined using the HPLC-ESI-MS/MS method, including 6 PPDs (G-Rb1, Rb2, Rb3, Rc, Rd, and Rg3), 7 PPTs (G-Re, Rf, Rg1, Rg2, Rh1, F3, and N-R1), and 3 OAs (G-Ro, CS-IV, and CS-IVa). The chromatograms obtained with reference substances and sample solutions are shown in Figure 4. The determination results are shown in Table 6 and Figure 5. In PJ, PM, and PZ, the contents of the PPDs were 4.449 ± 2.902%, 10.793 ± 6.135%, and 12.607 ± 4.247%, respectively. Among them, G-Ro, CS-IV, and CS-IVA have the highest content in plants, which is about 20–70 times that of other ginsenosides. Moreover, the content of G-Rb2, G-Rb3, G-RC, G-R3, and NG-R1 in plants is generally low, and some plants are almost undetectable. By analyzing the amount of compound, we can find that the difference in the content of these compounds (G-Rd, G-Rf, G-F3, G-Ro, and CS-IV) is larger than the others between PM and PJ. The differences in the content of NG-R1, G-Rb1, G-F3, G-Rh1, G-Re, G-Rg1, are larger than the others between PM and PZ. The differences in the content of G-Rh1, NG-R1, G-Rg1, G-F3, G-Ro, and CS-IVA between PJ and PZ are significant. In general, PJ and PM are similar, and PZ differs greatly from them.

The contents of PPTs were 6.091 ± 2.143%, 6.531 ± 2.544%, and 52.473 ± 7.064%. The contents of OAs were 184.357 ± 42.448%, 138.861 ± 20.353%, and 132.469 ± 17.248%. The contents of total ginsenosides were 194.897 ± 44.410%, 156.185 ± 19.814%, and 197.548 ± 27.227%, respectively. Notably, PZ has a higher level of PPTs content than PJ and PM (by more than 8 times). In addition, G-Ro, CS-IV, and CS-IVa were the three major ginsenosides in PJ and PM, whereas G-Ro, CS-IV, and G-Rg1 were the three major ginsenosides in PZ. Different types of ginsenosides possessed different pharmacological activities. Based on the results, all three drugs clearly contained a large number of OAs and a small amount of dammarane ginsenosides, especially PJ and PM. Furthermore, the content of ginsenosides differed greatly between the three drugs, which may be the reason for their differences in clinical application.

### 3.4. Discrimination of PJ, PM, and PZ by a Multivariate Statistical Analysis

The variations in the 16 major compounds among the three ginseng species were intuitively represented by a two-way hierarchical clustering analysis heat map. As shown in the heat map in Figure 5E, PZ could be clearly distinguished from PJ and PM by a hierarchical clustering analysis. In contrast, PJ and PZ were not well-discriminated, and their ginsenoside contents were much closer than that of PZ.

**Figure 6:**
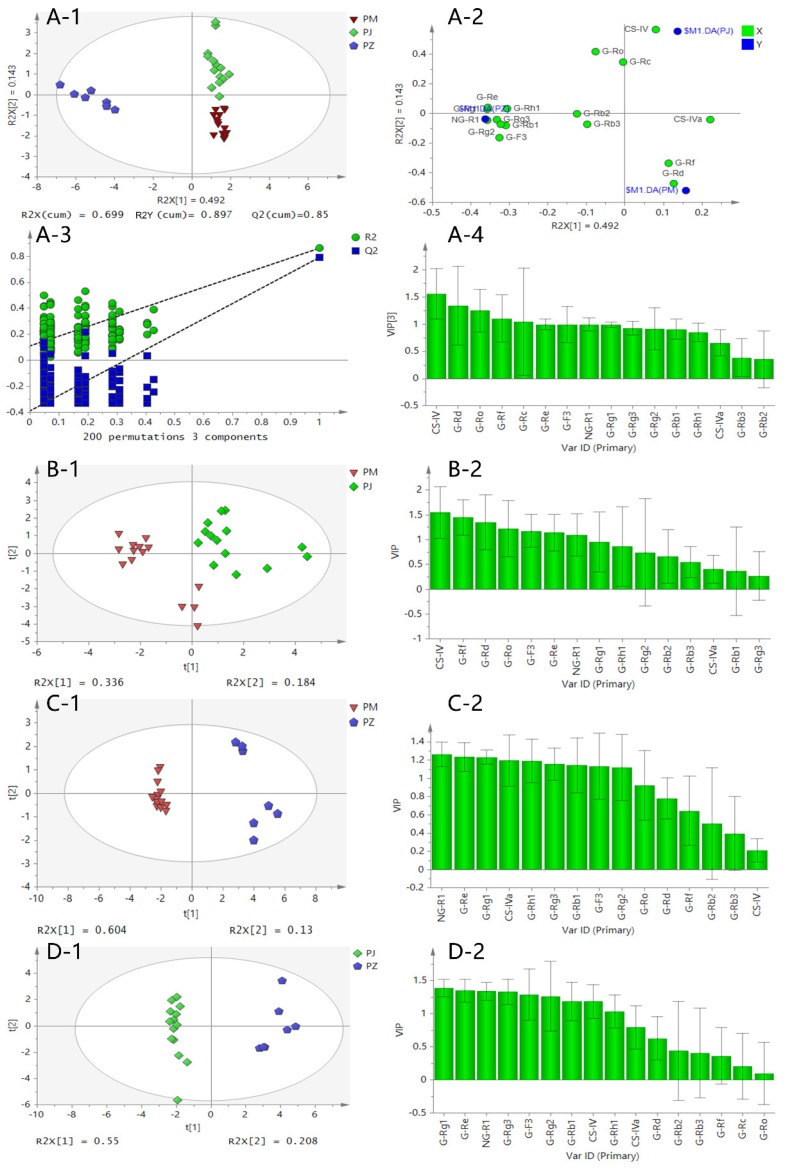
The PLS-DA (partial least squares–discriminate analysis) score scatter plot (**A**-**1**), the loading scatter plot (**A**-**2**), the chance permutation test to validate the occurrence or absence of over-fitting of the PLS-DA (partial least squares–discriminate analysis) model (**A**-**3**), the VIP (variable importance in the projection) values of the 16 saponins (**A**-**4**), the OPLS-DA (orthogonal partial least squares–discriminant analysis) score scatter plot based on a pairwise comparison (**B**-**1**, **C**-**1**, **D**-**1**), and the VIP values of the 16 saponins (**B**-**2**, **C**-**2**, **D**-**2**).

In order to further reveal differences in the chemical composition among PJ, PM, and PZ, PLS-DA and OPLS-DA were also utilized to distinguish between different ginseng species. A total of 50 ginseng samples (20 batches of PJ; 20 batches of PM; 10 batches of PZ) were analyzed. Among them, 35 samples were randomly selected as the training set, and 15 were the prediction set. The result is shown in Figure 6. The established PLS-DA model showed good fitness (R2X = 0.699, R2Y = 0.897) and predictability (Q2 = 0.85). The PLS-DA score plot displayed that the three clusters representing the PJ, PM, and PZ groups were well-segregated, thereby indicating the remarkable differences of ginsenosides among these three *Panax* herbs. A chance permutation test suggested that the model was not over-fitted (as shown in Figure 6A-3). All samples in the prediction set are correctly identified, so the PLS-DA model has a classification accuracy of 100%. The VIP (variable importance in the projection) plot was used to find the important compounds. When the VIP cutoff was set at 1.0, nine important compounds for discriminating between PJ, PM, and PZ were discovered. G-Rf, G-F3, and CS-IV have the highest contribution that can distinguish the above three herbs (as shown in Figure 6A-4).

The result of the OPLS-DA analysis based on pairwise comparison methods is shown in Figure 6B–D. The figure reveals that samples from the same species were tightly clustered together and that different species groups were discriminated from one another by the OPLS-DA score plot. Among those with a VIP value exceeding 1.0, seven important compounds for the discrimination between PJ and PM (in order of CS-IV, G-Rf, G-F3, G-Rd, G-Ro, G-Rb1, and G-Rg1), nine for PM and PZ (in order of G-Rh1, N-R1, G-F3, G-Rb1, CS-IVa, G-Rg1, G-Re, G-Rg3, and G-Rf), and nine for PJ and PZ (in order of G-Rh1, N-R1, G-Rg2, G-F3, G-Rg3, G-Rg1, G-Rb1, G-Re, and CS-IV) were obtained.

In Chinese folk medicine, PJ and PM are historically and generally used as a herbal medicine for similar indications. These two herbs possess combined medicinal effects, which are *P. ginseng*’s “conserving vitality” activities and *P. notoginseng*’s “replenishing blood” activities [10]. However, PJ and PM are considered to be two different herbal medicines, and are recorded as “zhujieshen” and “zhuzishen” in the Chinese Pharmacopoeia, respectively. Thus, investigation on the difference in their chemical constituents and biopharmalogical effects is necessary and crucial. In our qualitative and quantitative analyses based on an LC-MS technique, PLS-DA and OPLS-DA clearly distinguished between the two herbs, even though they are very similar in composition. Furthermore, the average content of total ginsenosides in PJ was conspicuously higher than that in PM, even though PM’s content in PPDs and PPTs was more abundant. These chemical differences may contribute to the differences in clinical application.

PZ, commonly known as ginger ginseng or Myanmar ginseng, is indigenous to Yunnan province in the Southwest of China [13], and has also been reported to wildly grow at the Par Moe Ne Water Spring area in Taung-gyi, Shan State, Myanmar, which has an altitude of 1500 m above sea level. Few previous studies have focused on the comprehensive chemical compositions and determination of ginsenosides in PZ [26]. In the present study, for the first time, the chemical compositions of this herb were screened and identified by UHPLC-Q-Exactive/HRMS, and the content of 16 major ginsenosides was determined. These results are beneficial for the development and quality assessment of PZ.

In conclusion, an integrated strategy to comprehensively identify the chemical composition and simultaneously quantify 16 ginsenodsies in the three *Panax* herbs was successfully established. After optimization of the conditions, a total of 101 ginsenosides were detected and tentatively identified using UHPLC-Q-Exactive/HRMS, including 82 from PJ, 78 from PM, and 67 from PZ. Among these compounds, 22 were unambiguously confirmed by comparing their retention times and mass spectra with those of reference ginsenosides. The quantitative analysis was implemented using a reliable and practical HPLC-ESI-MS/MS method using MRM mode. The validation of the methodology showed favorable levels of LOD, LOQ, linearity, precision, repeatability, stability, and recovery. Finally, the PLS-DA and OPLS-DA results, based on the quantitative data, displayed a significant difference in ginsenoside content between the three ginseng-drugs. G-Rf, G-F3, and CS-IV were the three most characteristic components that can distinguish between the three herbs. The integrated LC-MS-based strategy combined with a multivariate data analysis could not only achieve a rapid, accurate, and comprehensive qualitative and quantitative analysis of the complex ginsenoside but also enable us to discriminate between PJ, PM, and PZ, which provides valuable references for the quality assessment and control of traditional Chinese medicines (TCMs).

## Figures and Tables

**Table 1:** The sources of roots and rhizomes of *Panax japonicus* var. major (PM1–PM20), *Panax japonicas* (PJ1–PJ20), and *Panax zingiberensis* (PZ1–PZ10).

No.	Sample	Producing Area	Collection Time
1	PM-1	Hongyuan county, Sichuan province, China	2016
2	PM-2	Hongyuan county, Sichuan province, China	2016
3	PM-3	Yanyuan county, Sichuan province, China	2015
4	PM-4	Yanyuan county, Sichuan province, China	2015
5	PM-5	Wenchuan county, Sichuan province, China	2015
6	PM-6	Wenchuan county, Sichuan province, China	2015
7	PM-7	Kangding county, Sichuan province, China	2016
8	PM-8	Kangding county, Sichuan province, China	2016
9	PM-9	Kangding county, Sichuan province, China	2016
10	PM-10	Fugong county, Yunnan province, China	2015
11	PM-11	Fugong county, Yunnan province, China	2015
12	PM-12	Ludian county, Yunnan province, China	2015
13	PM-13	Ludian county, Yunnan province, China	2015
14	PM-14	Ludian county, Yunnan province, China	2015
15	PM-15	Nyingchi Prefecture, Tibet area, China	2015
16	PM-16	Nyingchi Prefecture, Tibet area, China	2015
17	PM-17	Nyingchi Prefecture, Tibet area, China	2015
18	PM-18	Nyingchi Prefecture, Tibet area, China	2015
19	PM-19	Bomi county, Tibet area, China	2015
20	PM-20	Bomi county, Tibet area, China	2015
21	PJ-1	En’shi city, Hubei province, China	2016
22	PJ-2	En’shi city, Hubei province, China	2016
23	PJ-3	En’shi city, Hubei province, China	2016
24	PJ-4	Xuan’en county, Hubei province, China	2016
25	PJ-5	Xuan’en county, Hubei province, China	2016
26	PJ-6	Leshan city, Sichuan province, China	2015
27	PJ-7	Leshan city, Sichuan province, China	2015
28	PJ-8	Meishan city, Sichuan province, China	2015
29	PJ-9	Meishan city, Sichuan province, China	2015
30	PJ-10	A’ba county, Sichuan province, China	2015
31	PJ-11	A’ba county, Sichuan province, China	2015
32	PJ-12	Ya’an City, Sichuan province, China	2015
33	PJ-13	Taibai county, Shaanxi province, China	2015
34	PJ-14	Taibai county, Shaanxi province, China	2015
35	PJ-15	Taibai county, Shaanxi province, China	2015
36	PJ-16	Taibai county, Shaanxi province, China	2015
37	PJ-17	Puer city, Yunnan province, China	2015
38	PJ-18	Puer city, Yunnan province, China	2015
39	PJ-19	Jinghong city, Yunnan province, China	2015
40	PJ-20	Wenshan county, Yunnan province, China	2015
41	PZ-1	Puer city, Yunnan province, China	2015
42	PZ-2	Puer city, Yunnan province, China	2015
43	PZ-3	Puer city, Yunnan province, China	2015
44	PZ-4	Puer city, Yunnan province, China	2015
45	PZ-5	Puer city, Yunnan province, China	2015
46	PZ-6	Puer city, Yunnan province, China	2015
47	PZ-7	Myanmar, Taung-gyi	2015
48	PZ-8	Myanmar, Taung-gyi	2015
49	PZ-9	Myanmar, Taung-gyi	2015
50	PZ-10	Myanmar, Taung-gyi	2015

**Table 2:** The retention time, precursor ions, product ions, and multiple reaction monitoring (MRM) parameters of the 16 analytes in the negative ion mode. Retention time (TR), declustering potential (DP), apillaryelectrophoresis (CE).

No.	Analyte	TR (min)	Precursor Ion (*m*/*z*)	Product Ion (*m*/*z*)	DP/V	CE/V
1	NG-R1	5.48	931.2	475.1	125	70
2	G-Rg1	6.42	799.2	637.1	100	55
3	G-Re	6.56	945.3	475.2	105	62
4	G-Rf	11.21	799.2	475.1	100	50
5	G-F3	12.09	769.2	475.1	105	55
6	G-Rg2	13.14	783.2	475.1	105	58
7	G-Rh1	13.27	637.2	475.1	75	45
8	G-Rb1	13.86	1107.2	945.1	115	65
9	G-Ro	14.68	955.1	793.0	110	60
10	G-Rc	14.72	1077.2	783.2	105	55
11	G-Rb2	15.70	1077.2	945.2	105	50
12	G-Rb3	16.01	1077.2	783.1	100	55
13	CS-IV	16.31	925.1	569.1	50	45
14	CS-IVa	17.48	793.2	569.2	35	45
15	G-Rd	17.51	945.2	621.2	105	60
16	G-Rg3	20.68	783.2	621.2	100	60

**Table 3:** Characterization of compounds using UPLC-Q-Exactive/MS (PJ, *Panax japonicas*; PM, *Panax japonicus* var. major; PZ, *Panax zingiberensis*).

No.	T_R_/min	Formula	[M − H]^−^		[M + HCOO]^−^	MS/MS	Identification	Sample
		**Calculated**	**Measured**	
1	5.07	C_53_H_88_O_23_	1091.56327	1091.52698		1023, 1007, 965, 783	Yesanchinoside G	PJ
2	5.31	C_48_H_82_O_19_	961.53666	961.53546	1007.54340	799, 781, 637, 619	G-Re1/G-Re2/G-Re3/NG-N/NG-M isomer	PJ, PZ
3	5.36	C_53_H_90_O_22_	1077.58400	1077.54590		945, 783, 637, 475, 391, 191	Floral G-M/Floral G-N	PJ, PM, PZ
4	5.44	C_53_H_88_O_23_	1091.56327	1091.52454		929, 767, 473	Yesanchinoside G isomer	PJ, PM, PZ
5	5.61	C_47_H_80_O_19_	947.52101	947.52203	993.52789	815, 653, 579, 491, 391	Vina G-R6/Yesanchinoside C	PZ
6	5.64	C_48_H_82_O_19_	961.53666	961.53775	1007.54224	799, 781, 637, 475, 391	20-glc-G-Rf	PJ, PM
7	5.74	C_48_H_82_O_19_	961.53666	961.53503	1007.54285	943, 931, 799, 637	G-Re1/G-Re2/G-Re3/NG-N/NG-M isomer	PJ, PM, PZ
8	5.75	C_54_H_92_O_23_	1107.59457	1107.59314	1153.60094	945, 783, 637, 475, 391	Yesanchinoside E	PM, PZ
9	5.92	C_41_H_70_O_14_	785.46818	785.46246	831.47443	739, 653, 491, 391	Majonoside R2 isomer	PJ
10	5.93	C_48_H_82_O_19_	961.53666	961.53602	1007.54651	799, 781, 637, 475, 391	20-glc-G-Rf isomer	PJ
11	5.94	C_53_H_90_O_22_	1077.58400	1077.58337	1123.58960	945, 783, 637, 475, 391, 191	FloralG-M/Floral G-N	PM
12	5.99	C_41_H_70_O_14_	785.46818	785.46429	831.47418	739, 653, 491, 391	Majonoside R2 isomer	PJ, PZ
13 *	6.01	C_47_H_80_O_18_	931.52609	931.52631	977.53192	799, 769, 637, 475	NG-R1	PJ, PM, PZ
14	6.09	C_42_H_72_O_14_	799.48383	799.48421		667, 653, 491, 455, 391	Pseudo G-F11 isomer	PJ, PM
15	6.10	C_51_H_82_O_18_	981.54174	981.54567		793, 763, 619, 581, 455, 371	Pseudo G-RT1 butyl ester	PM
16	6.11	C_56_H_94_O_24_	1149.60513	1149.60181	1195.61084	1149, 1107, 961, 783, 637, 475, 391	Acetyl Yesanchinoside E	PM
17 *	6.13	C_48_H_82_O_18_	945.54174	945.53772	991.54773	783, 637, 619, 475, 391, 205	G-Re	PJ, PM, PZ
18 *	6.18	C_42_H_72_O_14_	799.48383	799.48541	845.48975	637, 475, 391	G-Rg1	PJ, PM, PZ
19	6.27	C_45_H_74_O_17_	885.48423	885.48517		845, 829, 781, 637, 619, 475, 391	Malonyl-G-Rg1	PJ, PZ
20	6.29	C_58_H_98_O_26_	1209.62626	1209.59033		1165, 781, 619, 459	NG-Fc	PM
21	6.30	C_47_H_80_O_19_	947.52101	947.52191		815, 653, 579, 491, 391	Vina G-R6/Yesanchinoside C	PJ, PM, PZ
22	6.32	C_51_H_84_O_21_	1031.54214	1031.54419		987, 945, 637, 475, 391	Malonyl G-Re	PJ, PZ
23	6.34	C_41_H_70_O_14_	785.46818	785.46503	831.47430	739, 653, 491, 391	Majonoside R2 isomer	PJ, PM, PZ
24 *	6.40	C_42_H_72_O_14_	799.48383	799.48566	799.47870	754, 653, 491,473, 455	Pseudo G-F11	PJ, PM
25	6.46	C_48_H_82_O_19_	961.53666	961.53662	1007.54055	799, 781, 637, 499	G-Re1/G-Re2/G-Re3/NG-N/NG-M isomer	PJ, PM, PZ
26	6.53	C_50_H_84_O_19_	987.55231	987.55084	1033.55835	945, 791, 763, 637,475, 391, 275	Acetyl G-Re	PM, PJ, PZ
27	6.54	C_44_H_74_O_15_	841.49440	841.49204	887.50067	841, 795, 637, 475, 391	Acetyl-Rg1	PJ, PZ
28	6.55	C_42_H_72_O_15_	815.47875	815.47961	861.48315	637	Floralquinquenoside B	PJ, PM, PZ
29	6.60	C_48_H_82_O_19_	961.53666	961.53543	1007.54660	943, 799, 781, 457	G-Re1/G-Re2/G-Re3/NG-N/NG-M isomer	PJ
30	6.67	C_59_H_100_O_27_	1239.63682	1239.63452	1285.64172	1107, 1059, 945, 783, 621, 459	NG-R4/NG-Fa	PJ, PM, PZ
31	6.68	C_56_H_92_O_25_	1163.58439	1163.54858	1209.59326	1117, 955, 793, 621, 537, 459, 351	Malonyl G-Rc	PM
32	6.69	C_48_H_80_O_19_	959.52101	959.52148	1005.54272	797, 779, 635, 617, 473, 455	NG-G	PM, PZ
33	6.76	C_42_H_72_O_15_	815.47875	815.47610	861.48440	653, 491, 415	Floralquinquenoside D	PJ, PM
34	6.79	C_43_H_72_O_15_	827.47875	827.47998		695, 491, 455	Vina G-R2	PZ
35	6.82	C_58_H_98_O_26_	1209.62626	1209.62500	1255.63147	1077, 1047, 945, 783, 621, 459	G-Ra2	PJ, PM, PZ
36	6.84	C_63_H_106_O_30_	1341.66852	1341.66748	1387.67493	1209, 1077, 945, 783, 621, 459	NG-Q	PJ
37	6.86	C_44_H_74_O_15_	841.49440	841.49880	887.50098	799, 637, 475, 391	Acetyl-Rf	PJ, PM, PZ
38	6.89	C_59_H_100_O_27_	1239.63682	1239.63538	1285.64185	1077, 1059, 945, 783, 765, 459	NG-R4/NG-Fa	PJ, PM, PZ
39 *	6.92	C_54_H_92_O_23_	1107.59457	1107.59302	1153.59973	945, 783, 621, 459	G-Rb1	PJ, PM, PZ
40	6.95	C_57_H_94_O_26_	1193.59496	1193.59509		1149, 1107, 945, 783, 621, 459, 375	Malonyl G-Rb1	PJ, PM, PZ
41 *	6.96	C_42_H_72_O_14_	799.48383	799.48175	845.48956	637, 475, 391	G-Rf	PJ, PM, PZ
42	6.97	C_58_H_98_O_26_	1209.62626	1209.62488	1255.63074	1077, 1047, 915, 945, 783, 621, 459	G-Ra1	PJ, PZ
43	6.99	C_41_H_70_O_14_	785.46818	785.46852	831.47382	653, 491	Majonoside R2	PJ, PM, PZ
44	7.00	C_53_H_84_O_23_	1087.53196	1087.53149		955, 925, 793, 569, 497, 455, 283	Stipuleanoside R2	PJ, PM, PZ
45	7.03	C_61_H_100_O_29_	1295.62665	1295.62537		1251, 1209, 1077, 945, 783, 621, 459	Malonyl G-Ra2	PJ, PZ
46	7.04	C_42_H_72_O_14_	799.48383	799.48041	845.48962	637, 619, 499, 457	Majoroside F2/F3/F4	PJ, PM
47	7.04	C_48_H_76_O_19_	955.48971	955.49017	1001.49530	793, 631, 455, 349	G-Ro isomer	PJ, PM, PZ
48 *	7.05	C_53_H_90_O_22_	1077.58400	1077.58130	1123.58936	945, 783, 621, 459	G-Rc	PM, PZ
49	7.06	C_53_H_84_O_23_	1087.53196	1087.53235	1133.53625	925, 731, 569, 459	Stipuleanoside R2 isomer	PZ
50	7.07	C_56_H_92_O_25_	1163.58439	1163.58459		1119, 1077, 945, 783, 621, 459	Malonyl G-Rb2	PM
51	7.08	C_57_H_94_O_26_	1193.59496	1193.59497		1149, 1107, 945, 783, 621, 459, 375	Malonyl G-Rb1 isomer	PJ, PM
52 *	7.09	C_41_H_70_O_13_	769.47327	769.47125	815.47961	637, 485, 475, 325, 311	G-F3	PJ, PM, PZ
53	7.09	C_56_H_94_O_24_	1149.60513	1149.60083	1195.60669	1107, 945, 783, 621, 475	Yesanchinoside F	PJ, PM
54 *	7.15	C_53_H_90_O_22_	1077.58400	1077.58179	1123.58923	945, 915, 783, 621, 459	G-Rb2	PJ, PZ
55 *	7.16	C_48_H_76_O_19_	955.48971	955.48987	1001.49347	793, 731, 659,631, 455	G-Ro	PJ, PM, PZ
56	7.18	C_61_H_100_O_29_	1295.62665	1295.62561		1107, 945, 783, 621, 459	Malonyl G-Ra1	PJ
57	7.19	C_56_H_92_O_25_	1163.58439	1163.58435		1119, 1077, 945, 783, 621, 459, 293	Malonyl G-Rb3	PJ, PM
58 *	7.21	C_42_H_72_O_13_	783.48892	783.48627	829.49475	637, 619, 475, 457, 391, 205,161	G-Rg2	PJ, PM, PZ
59	7.22	C_45_H_74_O_17_	885.48423	885.48474		829, 799, 637, 475	Malonyl-G-Rf	PM
60 *	7.23	C_53_H_90_O_22_	1077.58400	1077.58289	1123.58960	945, 915, 783, 621, 459	G-Rb3	PZ
61 *	7.32	C_47_H_74_O_18_	925.47914	925.47894	971.48279	873, 793, 612, 569, 455	CS-IV	PJ, PZ
62 *	7.35	C_36_H_62_O_9_	637.43101	637.42828	683.43695	457, 391, 283, 255	G-Rh1	PJ, PM, PZ
63	7.37	C_48_H_82_O_19_	961.53666	961.53674	1007.54553	815, 781, 499	G-Re1/G-Re2/G-Re3/NG-N/NG-M isomer	PM
64 *	7.43	C_48_H_82_O_18_	945.54174	945.53973	991.54706	783, 621, 459	G-Rd	PJ, PM, PZ
65	7.44	C_51_H_84_O_21_	1031.54214	1031.54248		987, 945, 783, 621, 459, 375	Malonyl G-Rd	PJ, PM, PZ
66	7.46	C_50_H_84_O_19_	987.55231	987.55151	1033.55066	945, 783, 621, 459	Acetyl G-Rd	PM, PJ
67 *	7.53	C_42_H_66_O_14_	793.43688	793.43701	839.44269	631, 569, 509, 497, 455	CS-IVa	PJ, PM, PZ
68	7.60	C_51_H_84_O_21_	1031.54214	1031.54224		987, 945, 783, 621, 459, 375	Malonyl G-Rd isomer	PM, PJ
69 *	7.61	C_36_H_62_O_9_	637.43101	637.43042	683.43707	475, 391	G-F1	PJ, PM, PZ
70	7.63	C_48_H_82_O_18_	945.54174	945.53990	991.54767	899, 783, 855, 793, 621, 459	Gypenoside XVII	PJ, PM, PZ
71	7.64	C_53_H_90_O_23_	1093.57892	1093.57825		1047, 915, 783, 621, 459	Gypenoside LVI/LXVII/Floral G-P	PJ, PZ
72	7.66	C_49_H_78_O_19_	969.50536	969.99005	1015.51208	807, 631, 537, 455, 393	G-Ro methyl ester	PM, PZ
73	7.73	C_47_H_80_O_17_	915.53118	915.53667	961.53442	783, 621, 459, 375	NG-Fe	PJ, PM, PZ
74	7.79	C_38_H_64_O_10_	679.44157	679.44607		633, 611, 475,391	Acety G-Rh1	PJ, PM
75	7.82	C_47_H_80_O_17_	915.53118	915.52783	961.53748	783, 621, 459, 375	CS-III	PJ, PZ
76	7.82	C_48_H_82_O_19_	961.53666	961.53802	1007.53357	931, 799, 619	G-Re1/G-Re2/G-Re3/NG-N/NG-M isomer	PJ, PM, PZ
77	7.90	C_38_H_64_O_10_	679.44157	679.44049		633, 475, 391	Acety G-F1	PJ, PM
78	7.92	C_41_H_70_O_13_	769.47327	769.43805		679, 637, 475, 391	Pseudo G-RT3/G-F5	PM, PZ
79	7.94	C_48_H_82_O_19_	961.53666	961.53735	1007.54629	915, 621, 499	G-Re1/G-Re2/G-Re3/NG-N/NG-M isomer	PM, PJ, PZ
80	8.11	C_43_H_68_O_14_	807.45253	807.41650	853.46051	793, 631, 455	CS-IVa methyl ester	PJ, PM, PZ
81	8.17	C_42_H_66_O_14_	793.43688	793.43787	839.44141	631, 613, 569, 455	CS-II	PM, PZ
82	8.19	C_47_H_74_O_18_	925.47914	925.47986		793, 731, 569, 497, 455	CS-Ib	PJ, PM, PZ
83	8.31	C_47_H_74_O_18_	925.47914	925.47998	971.48346	793, 763, 631, 455	pseudo G-RT1/Stipuleanoside R1	PJ, PM, PZ
84	8.38	C_42_H_66_O_14_	793.43688	793.43707	839.44318	731, 631, 613, 569, 455	Zingibroside R1	PJ, PM, PZ
85 *	8.42	C_42_H_72_O_13_	783.48892	783.48669	829.49506	621, 459	(20*S*) G-Rg3	PM
86	8.67	C_41_H_64_O_13_	763.42632	763.42659	809.43291	631, 613, 569, 455, 325	Pseudo G-RP1	PJ, PM
87	8.68	C_42_H_70_O_12_	765.47835	765.43286		603, 593, 441	G-Rk1	PJ, PM, PZ
88 *	8.71	C_42_H_72_O_13_	783.48892	783.48826	829.49457	621, 459	G-F2	PJ, PM, PZ
89	8.78	C_42_H_72_O_13_	783.48892	783.48615	829.49524	752, 599, 459	(20R)-G-Rg3	PJ, PM, PZ
90	8.89	C_41_H_64_O_13_	763.42632	763.42631		613, 569, 497, 455, 405	Pseudo G-RP1 isomer	PJ, PM, PZ
91	8.90	C_42_H_70_O_12_	765.47835	765.43274		719, 673, 603, 573, 459	G-Rg5	PJ, PM, PZ
92	8.95	C_42_H_70_O_11_	749.48344	749.48045		617, 455	Pjs-4	PJ
93	9.11	C_58_H_96_O_24_	1175.62078	1175.67310	1221.67749	1159, 1095, 955, 793, 613, 569, 459	G-Ra6	PJ
94	9.64	C_37_H_62_O_7_	617.44118	617.44056	663.00000	455, 359	Oleanolic acid 28-*O*-β-D-glucopyranoside	PJ, PM, PZ
95	9.71	C_44_H_74_O_15_	841.49440	841.49487		795, 633, 491, 471	Vina G-R1	PJ, PM, PZ
96 *	9.84	C_30_H_52_O_4_	475.37819	475.36335		459, 391	PPT	PJ, PM, PZ
97	9.87	C_36_H_60_O_7_	603.42553	603.33807	649.34393	441, 279	G-Rk2	PJ, PM
98	10.08	C_36_H_62_O_8_	621.43610	621.43738	667.44208	459, 375, 325, 311	G-K	PJ, PM
99 *	10.18	C_36_H_62_O_8_	621.43610	621.43530	667.44232	459, 375, 283, 255	G-Rh2	PJ, PM, PZ
100	10.34	C_36_H_62_O_10_	653.42592	653.42682		491	Pseudo G-RT4	PJ, PM
101 *	10.94	C_30_H_52_O_3_	459.38327	459.39596		375, 329	PPD	PJ, PM

* identified with a standard reference.

**Table 4:** The regression equations, linear range, limit of detection (LOD), and limit of quantification (LOQ) of the 16 analytes.

No.	Analyte	Regression Equations	Correlation Coefficients (r)	Linear Range (ng × mL^−1^)	LOD (ng × mL^−1^)	LOQ (ng × mL^−1^)
1	N-R1	Y = 22.1X + 29.4	0.9991	1.56~1560	0.61	1.22
2	G-Rg1	Y = 7.7X + 193	0.9999	3.91~3910	1.91	3.81
3	G-Re	Y = 18X + 103	0.9998	6.25~6250	0.38	1.22
4	G-Rf	Y = 53.4X + 20	0.9998	0.78~780	0.19	0.38
5	G-F3	Y = 12.3X + 8.91	0.9997	1.17~1170	0.36	0.91
6	G-Rg2	Y = 71.7X + 50.6	0.9994	7.03~7030	0.21	0.86
7	G-Rh1	Y = 2.92X − 1.56	0.9992	6.24~1560	2.22	5.90
8	G-Rb1	Y = 7.5X − 105	0.9996	3.15~3130	1.53	3.05
9	G-Ro	Y = 27.8X + 3750	0.9992	9.38~9380	0.29	0.88
10	G-Rc	Y = 7.97X + 65.1	0.9998	1.56~1560	0.61	1.22
11	G-Rb2	Y = 12.3X + 78.7	0.9998	1.56~1560	0.41	1.22
12	G-Rb3	Y = 15.1X + 56.6	0.9996	1.56~1560	0.41	1.38
13	CS-IV	Y = 79.3X + 2610	0.9997	6.25~6250	0.16	0.31
14	CS-IVa	Y = 13.4X + 844	0.9991	7.81~7810	0.13	0.38
15	G-Rd	Y = 14X + 188	0.9993	1.56~1560	0.41	1.07
16	G-Rg3	Y = 24.1X − 17.1	0.9995	0.78~780	0.25	0.76

**Table 5:** The precision, repeatability, stability, and recovery of the 16 analytes.

No.	Analyte	Precision (RSD, %, n = 6)	Repeatability (RSD, %, n = 6)	Stability (RSD, %, n = 6)	Recovery	
					**Measured (%)**	**RSD (%)**
1	N-R1	1.78	3.12	4.43	99.25	3.45
2	G-Rg1	3.23	4.93	3.93	101.21	2.45
3	G-Re	3.65	2.86	4.43	103.19	0.94
4	G-Rf	4.87	4.05	4.60	99.92	2.74
5	G-F3	4.52	4.86	3.53	100.60	3.22
6	G-Rg2	1.32	4.19	3.79	101.40	1.90
7	G-Rh1	4.72	4.11	2.09	100.22	2.68
8	G-Rb1	4.59	4.10	2.02	104.10	0.69
9	G-Ro	2.99	1.40	1.02	100.38	2.84
10	G-Rc	4.16	2.90	2.60	102.00	2.37
11	G-Rb2	3.78	3.70	4.43	102.29	2.61
12	G-Rb3	2.29	4.26	4.56	102.67	1.56
13	CS-IV	2.70	4.32	2.47	100.98	2.68
14	CS-IVa	3.18	4.74	1.67	101.55	1.89
15	G-Rd	4.21	3.34	3.72	100.73	3.14
16	G-Rg3	2.72	3.41	3.31	101.67	2.77

RSD, relative standard deviation.

**Table 6:** The contents (mg × g^−1^) of the 16 ginsenosides in the rhizomes of *Panax japonicas* (PJ), *Panax japonicus* var. major (PM), and *Panax zingiberensis* (PZ).

Batch	PPD-Type Ginsenoside							PPT-Type Ginsenoside						OA-Type Ginsenoside			T_PPD_	T_PPT_	T_OA_	T
	G-Rb_1_	G-Rb_2_	G-Rb_3_	G-Rc	G-Rd	G-Rg_3_	NG-R_1_	G-Rg_1_	G-Re	G-Rf	G-F_3_	G-Rg_2_	G-Rh_1_	G-Ro	CS-IV	CS-IVa				
PM-1	5.104	0.240	0.221	0.037	2.320	0.053	0.039	1.888	3.328	2.816	2.480	1.424	0.298	55.200	46.240	29.760	7.976	12.273	131.200	151.448
PM-2	5.664	0.266	0.245	0.011	1.344	0.026	0.057	2.064	2.864	2.352	1.558	1.195	0.381	50.880	45.760	31.680	7.555	10.472	128.320	146.346
PM-3	4.016	0.149	0.160	0.016	0.958	-	0.041	0.786	1.984	1.744	1.302	0.925	0.159	53.920	49.440	31.584	5.300	6.941	134.944	147.185
PM-4	3.648	0.113	0.173	0.009	0.960	0.026	0.039	1.197	1.616	1.840	1.416	1.003	0.181	63.680	44.640	32.000	4.929	7.292	140.320	152.541
PM-5	5.488	0.246	0.259	0.042	1.184	0.054	0.025	1.022	2.464	2.096	1.824	1.158	0.151	61.760	41.120	43.040	7.273	8.740	145.920	161.934
PM-6	3.472	0.278	0.275	0.014	0.699	0.027	0.024	1.616	1.541	1.595	1.453	0.738	0.221	71.040	49.120	34.560	4.766	7.188	154.720	166.673
PM-7	2.944	0.109	0.158	0.009	0.765	0.056	0.089	1.726	2.080	1.515	1.320	0.483	0.162	82.080	27.520	40.960	4.040	7.375	150.560	161.975
PM-8	5.712	0.139	0.182	0.005	0.890	0.026	0.056	1.507	3.008	1.525	1.808	0.576	0.376	106.080	19.840	60.160	6.955	8.856	186.080	201.891
PM-9	3.232	0.080	0.118	0.008	0.539	-	0.078	1.352	4.096	1.603	2.624	0.392	0.317	104.800	27.840	54.240	3.977	10.462	186.880	201.319
PM-10	3.904	0.200	0.296	-	14.557	-	0.259	0.059	0.136	0.666	2.656	0.099	0.111	78.560	23.856	29.120	18.957	3.986	131.536	154.479
PM-11	4.256	0.250	0.231	-	13.240	0.067	0.291	0.026	0.166	0.518	2.688	0.013	0.130	81.600	22.338	33.440	18.044	3.832	137.378	159.253
PM-12	4.240	0.274	0.489	-	17.056	0.053	0.347	-	0.099	0.678	2.726	0.009	0.130	76.160	27.040	28.640	22.112	3.991	131.840	157.943
PM-13	4.736	0.168	0.386	-	15.824	0.026	0.293	-	0.163	0.929	3.086	0.058	-	78.880	27.200	26.400	21.140	4.530	132.480	158.150
PM-14	4.992	0.355	0.292	-	15.744	0.111	0.307	-	0.077	0.747	2.720	0.035	-	75.520	27.360	33.440	21.494	3.886	136.320	161.701
PM-15	3.296	0.255	0.296	-	5.894	-	0.235	0.421	0.714	0.208	3.936	0.451	0.062	91.200	11.360	35.680	9.741	6.027	138.240	154.008
PM-16	2.992	0.267	0.250	-	5.304	-	0.232	0.200	0.904	0.176	4.443	0.291	0.117	96.160	12.960	40.320	8.812	6.364	149.440	164.617
PM-17	2.464	0.330	0.354	-	7.968	0.040	0.206	0.331	0.387	0.136	3.376	0.205	0.090	65.120	6.128	46.080	11.155	4.732	117.328	133.215
PM-18	3.024	0.485	0.376	-	6.640	0.026	0.234	0.232	0.429	0.126	2.576	0.302	0.102	81.600	6.544	37.760	10.551	4.001	125.904	140.456
PM-19	1.984	0.354	0.224	-	6.848	0.036	0.261	0.378	0.338	0.068	3.504	0.192	0.107	75.840	5.216	30.880	9.445	4.847	111.936	126.228
PM-20	2.640	0.461	0.635	-	7.904	-	0.278	0.224	0.208	0.099	3.632	0.300	0.089	66.880	4.272	34.720	11.640	4.830	105.872	122.342
Mean	3.890	0.251	0.281	0.008	6.332	0.031	0.170	0.751	1.330	1.072	2.556	0.493	0.159	75.848	26.290	36.723	10.793	6.531	138.861	156.185
SD (n = 20)	1.110	0.110	0.122	0.012	5.925	0.029	0.116	0.722	1.277	0.851	0.926	0.437	0.109	15.590	15.537	8.686	6.135	2.544	20.353	19.814
PJ-1	1.626	0.061	-	-	0.355	0.031	0.055	5.360	2.016	0.422	0.418	0.136	-	88.480	50.720	10.880	2.073	8.407	150.080	160.560
PJ-2	1.280	-	-	-	0.352	0.029	-	3.232	2.432	0.650	0.789	0.189	0.130	101.120	37.440	10.544	1.661	7.422	149.104	158.186
PJ-3	1.363	-	-	0.010	0.432	0.054	-	2.128	1.427	1.099	0.571	0.131	0.130	70.080	39.680	8.816	1.859	5.487	118.576	125.922
PJ-4	1.280	0.009	-	-	0.917	0.004	0.010	2.992	1.760	0.512	0.922	0.158	-	77.280	33.600	-	2.210	6.354	120.880	129.443
PJ-5	1.499	-	0.028	-	0.701	0.056	-	4.560	1.760	0.638	0.944	0.136	-	77.600	31.840	5.984	2.284	8.039	115.424	125.746
PJ-6	7.728	0.406	0.549	-	1.648	-	0.034	0.669	1.619	0.020	2.642	0.274	-	141.280	49.280	54.880	10.331	5.257	245.440	261.028
PJ-7	9.232	0.219	0.416	-	1.363	0.041	0.036	0.882	2.336	0.033	2.762	0.318	-	139.200	45.920	55.040	11.272	6.366	240.160	257.798
PJ-8	2.064	0.328	0.378	-	1.624	0.006	0.145	0.294	1.274	0.037	2.896	0.432	-	99.520	48.160	46.400	4.400	5.078	194.080	203.558
PJ-9	1.856	0.674	0.507	-	1.792	0.026	0.087	0.394	2.064	0.019	2.752	0.434	-	95.840	58.400	39.200	4.855	5.750	193.440	204.044
PJ-10	2.064	0.248	0.253	0.099	1.574	0.040	0.130	2.192	1.421	0.012	1.856	0.296	0.162	88.800	39.360	38.080	4.278	6.068	166.240	176.587
PJ-11	5.456	0.246	0.276	0.054	1.512	0.097	0.132	1.680	0.736	0.032	2.338	0.190	0.115	118.880	41.600	44.640	7.642	5.222	205.120	217.984
PJ-12	5.792	0.707	0.320	0.038	2.256	0.026	0.055	0.072	0.045	0.011	1.640	0.164	0.091	145.600	34.240	69.600	9.140	2.079	249.440	260.658
PJ-13	1.114	0.126	0.173	0.300	0.187	0.026	0.070	3.904	3.008	0.302	0.334	0.067	0.584	137.920	57.280	16.160	1.926	8.270	211.360	221.556
PJ-14	1.541	0.157	0.144	0.250	0.166	0.013	0.087	2.688	4.112	0.260	0.333	0.076	0.261	145.600	62.880	13.936	2.270	7.816	222.416	232.503
PJ-15	2.112	0.215	0.117	0.277	0.514	0.026	0.187	4.032	3.808	0.230	0.475	0.128	0.290	146.240	59.840	10.528	3.260	9.151	216.608	229.019
PJ-16	1.595	0.275	0.294	0.328	0.664	0.026	0.187	4.624	3.968	0.296	0.627	0.166	0.290	145.280	65.440	15.808	3.183	10.159	226.528	239.870
PJ-17	3.146	0.434	0.248	0.171	1.216	0.026	0.121	0.469	1.309	0.031	1.376	0.656	-	74.080	39.360	54.720	5.240	3.962	168.160	177.363
PJ-18	2.496	0.328	0.115	-	1.080	0.041	0.158	0.298	1.062	0.019	0.869	0.418	-	76.960	46.080	54.560	4.061	2.823	177.600	184.484
PJ-19	2.269	0.166	0.181	0.018	0.981	-	0.117	0.290	2.224	0.031	0.832	0.458	-	78.080	47.040	36.480	3.614	3.951	161.600	169.166
PJ-20	1.680	0.326	0.202	0.037	1.152	0.028	0.234	0.989	0.768	0.013	1.424	0.598	0.130	85.760	37.280	31.840	3.425	4.156	154.880	162.461
Mean	2.860	0.246	0.210	0.079	1.024	0.030	0.092	2.087	1.957	0.234	1.340	0.271	0.109	106.680	46.272	31.405	4.449	6.091	184.357	194.897
SD (n = 20)	2.310	0.203	0.168	0.116	0.597	0.022	0.069	1.730	1.093	0.299	0.898	0.173	0.152	29.528	10.162	20.349	2.902	2.143	42.448	44.410
PZ-1	9.136	0.178	0.029	0.014	0.450	0.450	3.488	24.640	10.960	0.058	10.080	2.912	3.984	93.760	25.600	11.472	10.256	56.122	130.832	197.209
PZ-2	8.128	0.161	0.053	0.020	0.453	0.331	3.760	21.760	10.128	0.079	10.112	2.992	2.880	91.520	25.760	10.944	9.146	51.711	128.224	189.082
PZ-3	8.464	0.142	0.034	0.011	0.451	0.222	3.808	20.320	8.768	0.080	9.392	2.944	2.736	86.080	25.120	10.560	9.324	48.048	121.760	179.133
PZ-4	8.256	0.071	0.053	0.020	0.515	0.328	3.888	17.040	8.736	0.050	9.712	3.376	2.160	80.960	23.840	9.744	9.244	44.962	114.544	168.750
PZ-5	8.288	0.105	0.118	0.013	0.421	0.245	3.872	16.160	9.440	0.067	11.552	3.440	1.696	84.160	23.840	12.224	9.190	46.227	120.224	175.641
PZ-6	9.296	0.213	0.007	0.015	0.541	0.290	4.768	16.160	9.376	0.095	10.480	3.344	2.720	88.640	25.600	12.224	10.361	46.943	126.464	183.768
PZ-7	10.528	0.762	0.789	0.117	0.374	0.478	3.808	22.085	14.720	0.056	5.536	0.819	5.184	99.680	22.080	11.792	13.048	52.208	133.552	198.808
PZ-8	14.720	1.378	1.286	0.197	1.168	0.371	3.568	22.402	15.808	0.039	4.786	1.619	3.008	89.920	18.240	15.120	19.120	51.229	123.280	193.629
PZ-9	15.840	0.408	0.397	0.037	0.352	0.413	5.440	22.254	15.344	0.093	10.560	2.128	3.424	118.080	28.000	9.872	17.447	59.243	155.952	232.642
PZ-10	17.120	0.272	0.507	0.144	0.496	0.390	5.792	26.244	16.960	0.048	12.688	2.352	3.952	125.600	29.440	14.816	18.930	68.036	169.856	256.821
Mean	10.978	0.369	0.327	0.059	0.522	0.352	4.219	20.907	12.024	0.066	9.490	2.593	3.174	95.840	24.752	11.877	12.607	52.473	132.469	197.548
SD (n = 10)	3.510	0.408	0.427	0.068	0.234	0.084	0.817	3.479	3.280	0.020	2.476	0.859	1.005	14.750	3.097	1.848	4.247	7.064	17.248	27.227

T_PPD_, T_PPT_, T_OA_, and T represent the content of protopanaxdiol-type ginsenosides, protopanaxtriol-type ginsenosides, oleanolic acid-type ginsenosides, and total ginsenosides, respectively; “-” represents not detected; SD, standard deviation.
